# Longitudinal analysis of long-term outcomes of abdominal flap-based microsurgical reconstruction and two-stage prosthetic reconstruction

**DOI:** 10.1038/s41598-023-31218-2

**Published:** 2023-03-11

**Authors:** Kyeong-Tae Lee, Jina Kim, Byung Joon Jeon, Jai Kyong Pyon, Sa Ik Bang, Goo-Hyun Mun

**Affiliations:** Department of Plastic Surgery, Samsung Medical Center, Sungkyunkwan University of Medicine, 81 Irwon-Ro, Gangnam-Gu, Seoul, 06351 South Korea

**Keywords:** Oncology, Risk factors, Signs and symptoms

## Abstract

Two-stage tissue expander/implant (TE/I) and deep inferior epigastric perforator (DIEP) flaps are the two main pillars of breast reconstruction. This study aimed to conduct a longitudinal analysis of long-term outcomes after immediate DIEP- and TE/I-based reconstruction. This retrospective cohort study included patients with breast cancer who underwent immediate DIEP- or TE/I-based reconstruction between 2012 and 2017. The cumulative incidence of major complications, defined as unplanned reoperation/readmission due to complications, was analyzed by the reconstruction modality and its independent association. In total, 1,474 cases (1,162 TE/I and 312 DIEP cases) were analyzed, with a median follow-up of 58 months. The 5-year cumulative incidence of major complications was significantly higher in the TE/I group (10.3% vs. 4.7%). On the multivariable analyses, the use of DIEP flap was associated with a significantly reduced risk of major complications compared to that of TE/I. A more prominent association was observed in the analysis of patients who received adjuvant radiotherapy. Restricting analysis to those who received adjuvant chemotherapy revealed no differences between the two groups. The rate of reoperation/readmission for improving aesthetic outcomes was similar in the two groups. Long-term risks for unexpected reoperation/readmission may differ between DIEP- and TE/I-based immediate reconstruction.

## Introduction

Breast reconstruction has become an essential pillar in breast cancer treatment, owing to its established advantages in improving self-esteem, psychosocial well-being, and quality of life^1,2^. Accordingly, the global rate of immediate breast reconstruction following total mastectomy has steeply increased. Currently, there are two mainstream breast reconstruction modalities: autologous flap reconstruction and prosthesis-based reconstruction. Specifically, the most commonly used methods of the former and latter types are deep inferior epigastric artery perforator (DIEP) flaps, and two-stage tissue expander/implant-based (TE/I) method, respectively. Each method has its own pros and cons, and the most appropriate method may differ by individuals. Reconstructive surgeons select the best modality for each patient based on a variety of factors, including its safety representatively.

Surprisingly, despite the long-standing, prevalent use of these two methods, the method that provides safer and more reliable outcomes with a lower rate of postoperative morbidities remains unclear. Several clinical investigations compared postoperative complications between the two methods^3–7^. However, most were limited by their cross-sectional study design, which lacked analyses with time passage. Recently, several longitudinal studies have assessed the development of complications following breast reconstruction and compared them among diverse methods, including DIEP flaps and the two-stage TE/I method^6–8^. However, these studies did not reach consistent conclusions regarding which method could be safer with a lower cumulative incidence of complications. Despite their elaborate study designs and relatively large sample sizes, these studies had a relatively short follow-up period of 2–3 years, which might have led to inconsistent results. With advancements in cancer treatment, breast cancer survivors may have a long life expectancy, and a reconstructed breast could be a lifelong companion for these patients. In addition, adjunct treatment of breast cancer, including radiotherapy and/or chemotherapy, which may influence the outcomes of breast reconstruction, has been sustained for years. Therefore, the outcomes of breast reconstruction need to be analyzed over longer periods. Debates on the long-term safety of the two representative methods for breast reconstruction are still ongoing with lack of relevant evidence.

This study aimed to investigate the longitudinal outcomes of immediate breast reconstruction using DIEP flaps and two-stage TE/I for a longer follow-up period, to compare the risks of adverse outcomes between the two modalities, and to determine whether the relative risks of the two methods differ according to specific oncologic treatment settings.

## Methods

### Study participants

Patients with breast cancer who underwent immediate breast reconstruction after total mastectomy between 2012 and 2017 were retrospectively reviewed. Patients who had undergone DIEP flap surgery (DIEP flap group) or two-stage TE/I for breast reconstruction (TE/I group) were included. The following patients were excluded: those who used other reconstruction modalities, such as latissimus dorsi myocutaneous or direct-to-implant flaps, which were rarely adopted in our institution during the study period; those who did not complete both stages during the follow-up period owing to causes unrelated to complications; those who underwent the second stage of exchange-to-implant in another clinic; those who had planned conversion to autologous tissue reconstruction following tissue expander insertion; and those who had been lost to follow-up within 2 years postoperatively.

In the authors’ institution, the reconstruction modality was determined by attending reconstructive surgeons, mainly considering patients body habitus and desires. Generally, for patients having higher BMI or large and ptotic breasts, the use of DIEP flap was preferentially considered. Patients’ oncologic conditions including possibility of receiving adjuvant radiotherapy or chemotherapy were not a primary consideration in choosing reconstruction modality.

This study was conducted in accordance with the principles of the Declaration of Helsinki. The study was approved by the institutional review board of Samsung Medical Center (IRB No. 2022-07-041). The requirement for informed consent was waived owing to the retrospective nature of the study.

### Data collection and outcome measurements

Data regarding patient characteristics (age, body mass index [BMI], comorbidities), operation (type of mastectomy, weight of the mastectomy specimen, type of reconstruction), and adjunct treatment-related characteristics (neoadjuvant chemotherapy, adjuvant chemotherapy, adjuvant radiotherapy) were collected from our prospectively maintained database, which was regularly updated by ancillary doctors.

Data regarding the development of postoperative complications could have been underestimated due to the retrospective study design and the underreporting of adverse events in medical charts. Therefore, we conducted a subsequent analysis focusing on the development of reoperation or readmission, wherein data were accurately recorded in the medical charts and were subject to the least risk of underestimation.

The primary outcome of interest was major complications, defined as unexpected reoperations under general anesthesia due to postoperative complications, which were conducted beyond the index reconstructive operations, and/or unexpected readmission due to postoperative complications. The index reconstructive operations included primary reconstruction (transfer of abdominal flaps or insertion of a tissue expander), the second stage of exchange-to-implant for cases of prosthetic reconstruction, reconstruction of the nipple-areolar complex, and tattooing. The development of reconstruction failure, which was defined as flap removal or TE/I and/or unplanned conversion to other modalities, was also noted and analyzed. The secondary outcomes were reoperations under general anesthesia or readmission for improving aesthetic outcomes or increasing patient satisfaction, which were unrelated to postoperative complications.

The date and type of adverse events (unplanned reoperation, readmission, or reconstruction failure) and the date of the last follow-up were documented. The time until the development of adverse events was calculated and used to assess the event-free duration.

### Statistical analysis

The association between categorical variables of interest was evaluated using Pearson’s chi-squared test or Fisher’s exact test. Analyses between continuous and categorical variables were conducted using the Student’s t-test or the Mann–Whitney U test. The Kaplan–Meier method was used to calculate the cumulative incidence of major complications or reconstruction failure, for which differences were compared using the log-rank test. The endpoint analyzed was the development of adverse outcomes during the follow-up period. Cases were censored if the respective events had not occurred during the follow-up period. Univariate and multivariate Cox regression analyses were conducted to identify independent predictors of outcomes with hazard ratios (HRs) and the corresponding 95% confidence intervals (CIs). For multivariate analyses, the backward selection model was used with variables that *p-*values were < 0.01 in univariate analyses.

To adjust for potential heterogeneity in baseline characteristics between the two groups, propensity score matching analyses were conducted for 10 variables: age, BMI, diabetes, hypertension, smoking status, type of mastectomy, weight of the mastectomy specimen, neoadjuvant chemotherapy, adjuvant chemotherapy, and adjuvant radiotherapy. Based on the calculated propensity scores, the DIEP flap and TE/implant groups were matched at a ratio of 1:1, and the rates of adverse outcomes were compared. Univariate and multivariate analyses were also conducted after propensity score matching. All analyses were performed using SPSS version 20 (IBM Corporation, Armonk, NY, USA), and statistical significance was set at *p *< 0.05.

## Results

During the study period, 11,927 patients with breast cancer were treated with a partial or total mastectomy at our institution. Based on the above selection criteria, 1,474 cases representing 1,380 patients were finally included and analyzed. There were 1,286 unilateral and 94 bilateral reconstructions. Their mean age and BMI were 44.1 years (range, 18–66 years), and 22.5 kg/m^2^ (range, 15.1–39.8), respectively.

Of these, 1,162 underwent a two-stage prosthetic-based method, and 312 underwent a DIEP flap operation. Table 1 lists the baseline characteristics of the two groups. The DIEP flap group had an older age, a higher BMI, a greater weight of mastectomy specimen, and a lower rate of nipple-sparing mastectomy than the TE/I group. The rate of cases receiving neoadjuvant chemotherapy was higher in the DIEP flap group. Other characteristics, including comorbidities and adjuvant treatment, were similar between the groups. The median follow-up period was 58 months (range, 6–110 months).

**Table 1:** Comparison of baseline characteristics between DIEP flap versus TE/I groups.

	TE/I group	DIEP flap group	*p-*value
Patient No	1,078	302	
Case No	1,162	312	
Laterality (per patient)			
Unilateral	994 (95.9%)	292 (96.7%)	
Bilateral	84 (4.1%)	10 (3.3%)	
Age, mean (± SD), (yrs)	43.6 (± 7.5)	46.3 (± 6.6)	< 0.001
BMI, mean (± SD), (kg/m^2^)	22.2 (± 2.9)	23.6 (± 3.4)	< 0.001
Normal weight	925 (79.6%)	212 (67.9%)	< 0.001
Underweight	61 (5.2%)	9 (2.9%)	
Overweight/Obesity	176 (15.1%)	91 (29.2%)	
Diabetes	14 (1.2%)	5 (1.6%)	0.580
Hypertension	58 (5.0%)	25 (8.0%)	0.040
Smoking	19 (1.6%)	7 (2.2%)	0.468
Type of mastectomy			< 0.001
Nipple-sparing	327 (28.1%)	39 (12.5%)	
Skin-sparing	835 (71.9%)	273 (87.5%)	
Mastectomy weight, mean (± SD), (gram)	393.7 (± 216.2)	479.9 (± 206.1)	< 0.001
Neoadjuvant chemotherapy			0.029
Received	83 (7.1%)	34 (10.9%)	
Not received	1082 (92.9%)	278 (89.1%)	
Adjuvant chemotherapy			0.106
Received	449 (38.6%)	105 (33.7%)	
Not received	713 (61.4%)	207 (66.3%)	
Adjuvant radiotherapy			0.450
Received	223 (19.2%)	54 (17.3%)	
Not received	939 (80.8%)	258 (82.7%)	

**Figure 1:**
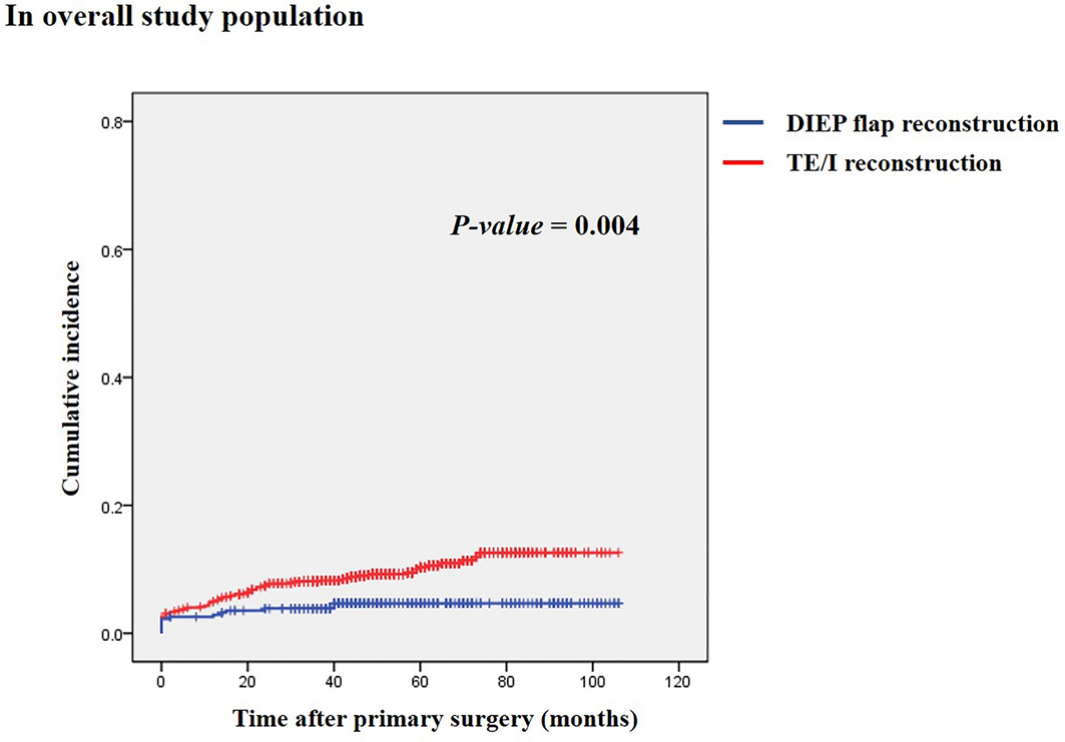
Comparison of cumulative incidence of major complications between the two groups using Kaplan–Meier analysis.

During the follow-up period, the major complications developed in 126 cases, including 94 reoperations conducted under general anesthesia and 32 with readmission only (see Table 2). The TE/I group had a significantly higher rate of the major complications (9.6% vs. 4.5%, *p *= 0.004). The distribution of the major complications according to the postoperative timing (≤ 2 months, 2 months to 2 years, or > 2 years) differed between the two groups. The rate of the major complications ≤ 2 months postoperatively was similar between the groups, whereas those from 2 months to 2 years and > 2 years postoperatively were significantly higher in the TE/I group. The 2- and 5-year cumulative incidences of the major complications and reconstruction failure rates were also higher in the TE/I group. In the Kaplan–Meier analysis, the TE/I group showed a significantly higher cumulative incidence of the major complications than the DIEP flap group (*p *= 0.004; see Fig. 1). The rates of reoperation and/or readmission for improving aesthetic outcomes did not differ between the two groups (*p *= 0.273).

**Table 2:** Comparison of outcomes between two groups.

Complications	TE/I group	DIEP group	*p-*value
Major complication	112 (9.6%)	14 (4.5%)	0.004
Type			
Infection	43 (3.7%)	2 (0.6%)	
Hematoma	15 (1.3%)	3 (1.0%)	
Extensive wound problem	0	1 (0.3%)	
Flap failure	0	2 (0.6%)	
Fat necrosis excision*	0	4 (1.3%)	
Abdominal wall weakness	0	2 (0.6%)	
Prosthesis failure (rupture or leakage)	13 (1.1%)	0	
Capsular contracture	19 (1.6%)	0	
Unplanned conversion to other modalities	22 (1.9%)	0	
Timing			0.019
Developed within postop 2 months	39 (3.4%)	8 (2.6%)	
Developed within postop 2 years	47 (4.0%)	4 (1.3%)	
Developed beyond postop 2 years	26 (2.2%)	2 (0.6%)	
2-year cumulative incidence	7.4%	3.9%	
5-year cumulative incidence	10.3%	4.7%	
Reconstruction failure	64 (5.5%)	2 (0.6%)	< 0.001
Timing			0.002
Developed within postop 2 months	14 (1.2%)	2 (0.6%)	
Developed within postop 2 years	39 (3.4%)	0	
Developed beyond postop 2 years	11 (0.9%)	0	
Reoperation/Readmission for aesthetic purpose	16 (1.4%)	7 (2.2%)	0.273

All analyses were conducted with the first event occurred during the follow-up period.

*The fat necrosis was a kind of partial flap necrosis that could be derived from insufficient perfusion to the flap adipose tissue. Excision of fat necrosis was conducted in patients who developed palpable nodules over 2 cm in diameter on their reconstructed breasts and wanted to remove the lesions.

In the univariate Cox regression analyses, the reconstruction method showed a significant association with the development of the major complications which remained significant after adjusting for other variables in the multivariate analyses. That is, the use of DIEP was associated with a significantly reduced incidence of the major complications compared to that of TE/I (HR, 0.415; 95% CI, 0.238–0.724; adjusted *p *= 0.002). Hypertension, the weight of the mastectomy specimen, and adjuvant radiotherapy were also significantly associated with the development of the major complications (see Table 3).

**Table 3:** Univariable and multivariable Cox-regression analysis for major complication.

	Univariable analysis		Multivariable analysis	
	HR (95% CI)	*p-*value	HR (95% CI)	*p-*value
Age	1.020 (0.996–1.045)	0.104		
BMI	1.073 (1.020–1.128)	0.006		
Diabetes	2.154 (0.685–6.774)	0.189		
Hypertension	2.183 (1.253–3.806)	0.006	2.108 (1.184–3.752)	0.011
Smoking	0.048 (0–16.919)	0.311		
Type of mastectomy		0.058		
Skin-sparing	Ref			
Nipple-sparing	1.435 (0.988–2.083)			
Mastectomy weight	1.001 (1.000–1.002)	< 0.001	1.001 (1.000–1.002)	0.004
Reconstruction method		0.005		0.002
TE/I	Ref		ref	
DIEP flap	0.448 (0.257–0.781)		0.415 (0.238–0.724)	
Neoadjuvant chemotherapy		0.604		
Not conducted	ref			
Conducted	0.827 (0.404–1.694)			
Adjuvant chemotherapy		0.037		
Not conducted	ref			
Conducted	1.451 (1.023–2.060)			
Adjuvant radiotherapy		< 0.001		< 0.001
Not conducted	ref		ref	
Conducted	2.451 (1.699–3.535)		2.296 (1.580–3.337)	

*HR* Hazard ratio, *CI* Confidence interval, *BMI* Body mass index, *ref* reference.

In multivariate analyses, the use of DIEP was also significantly associated with a decreased incidence of reconstruction failure compared to that of TE/I (HR, 0.105; 95% CI, 0.026–0.428).

### Analysis after propensity score matching

Further analyses were conducted on 312 pairs (624 cases) using propensity score matching. The two groups were successfully matched for all baseline characteristics, including age, BMI, co-morbidity, type of mastectomy, and adjuvant oncologic treatments (see Supplementary Table S1). Similar to the above analyses, the cumulative incidence of the major complications was significantly higher in the TE/I group than in the DIEP flap group (*p *= 0.027).

Multivariate analyses showed that the use of TE/I-based was significantly associated with an increased risk of developing the major complications (HR, 0.510; 95% CI, 0.266–0.977; adjusted *p *= 0.042).

### Subgroup analyses: According to adjuvant treatments

#### Adjuvant radiotherapy

**Figure 2:**
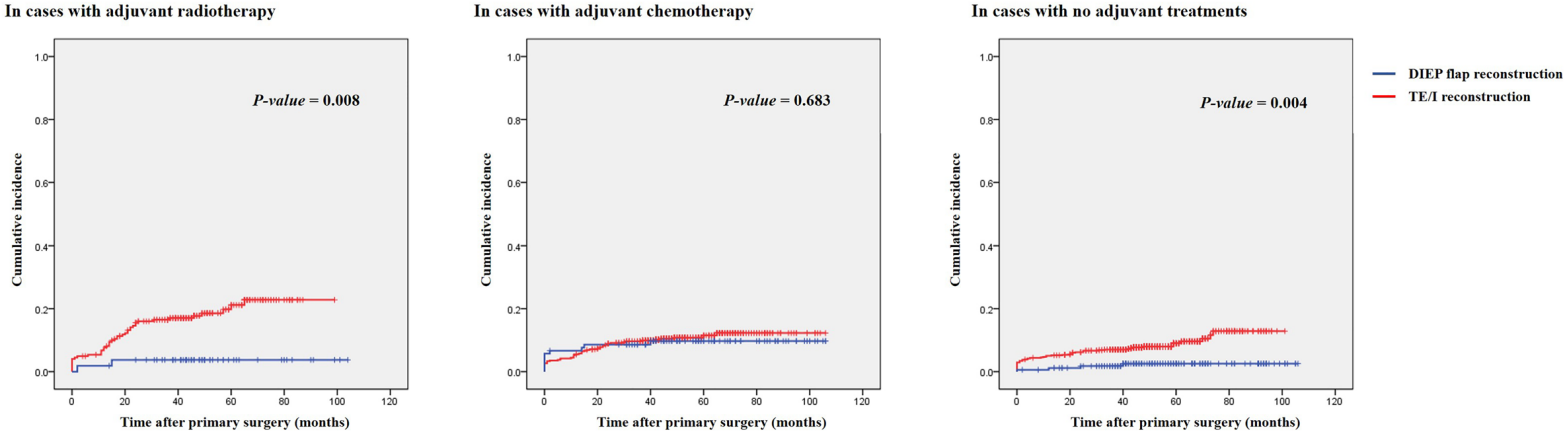
Comparison of the cumulative incidence of major complications between the two groups in diverse clinical situations according to adjuvant oncologic treatments in cases receiving (left) adjuvant radiotherapy, (middle) adjuvant chemotherapy, or (right) no adjuvant treatments.

To adjust for the potential confounding effects of adjunct oncologic treatments on the development of postoperative adverse events, subgroup analyses were performed. In the analysis of 277 patients receiving adjuvant radiotherapy, the rate of the major complications was significantly higher in the TE/I group, which was more prominent in the postoperative period between 2 months and 2 years (see Table 4). Kaplan–Meier analysis showed a significantly increased cumulative incidence of adverse events in the TE/I group throughout the follow-up period (*p *= 0.008; see Fig. 2). Multivariate analyses demonstrated that the reconstruction method was associated with the development of the major complications after adjusting for other variables, indicating that the TE/I method was associated with an increased rate of the major complications (see Supplementary Table S2).

**Table 4:** Comparison of cumulative incidence of major complications between two groups according to adjuvant oncologic treatments.

	TE/Implant group	DIEP flap group	*p-*value
In cases of adjuvant radiotherapy	223	54	
Major complication	42 (18.8%)	2 (3.7%)	0.006
Timing			0.053
Developed within postop 2 months	11 (4.9%)	1 (1.9%)	
Developed within postop 2 years	23 (10.3%)	1 (1.9%)	
Developed beyond postop 2 years	8 (3.6%)	0	
2-year cumulative incidence	14.6%	3.7%	
5-year cumulative incidence	21.2%	3.7%	
In cases of adjuvant chemotherapy	449	105	
Major complication	49 (10.9%)	10 (9.5%)	0.678
Timing			0.198
Developed within postop 2 months	16 (3.6%)	7 (6.7%)	
Developed within postop 2 years	24 (5.3%)	2 (1.9%)	
Developed beyond postop 2 years	9 (2.0%)	1 (1.0%)	
2-year cumulative incidence	9.0%	8.6%	
5-year cumulative incidence	11.6%	9.7%

#### Adjuvant chemotherapy

Among the 554 patients who had received adjuvant chemotherapy, the rate of the major complications was similar between the two groups. In the Kaplan–Meier analysis, the two groups showed similar cumulative incidences of adverse outcomes. However, the rate in the DIEP flap group was higher within two postoperative years, while that of the TE/I group exceeded that of the DIEP flap group in the long-term (*p *= 0.683; see Fig. 2). Consistent results were also observed in multivariate analyses, showing that the reconstruction method was not associated with the development of adverse outcomes in this subgroup. The weight of the mastectomy specimen and receiving adjuvant radiotherapy were significantly associated with the development of the major complications (see Supplementary Table S2).

#### No adjuvant treatment

A total of 806 patients did not receive any adjunct oncological treatment. Kaplan–Meier analysis showed a significantly higher cumulative incidence of the major complications in the TE/I group (*p *= 0.004; see Fig. 2). Consistently, multivariate analyses demonstrated that the DIEP flap group was associated with a significantly decreased rate (approximately one-fourth of the risk) of the major complications compared to the TE/I group after adjusting for other variables (see Supplementary Table 2).

### Subgroup analyses: According to reconstruction methods

#### In DIEP flap reconstruction

**Figure 3:**
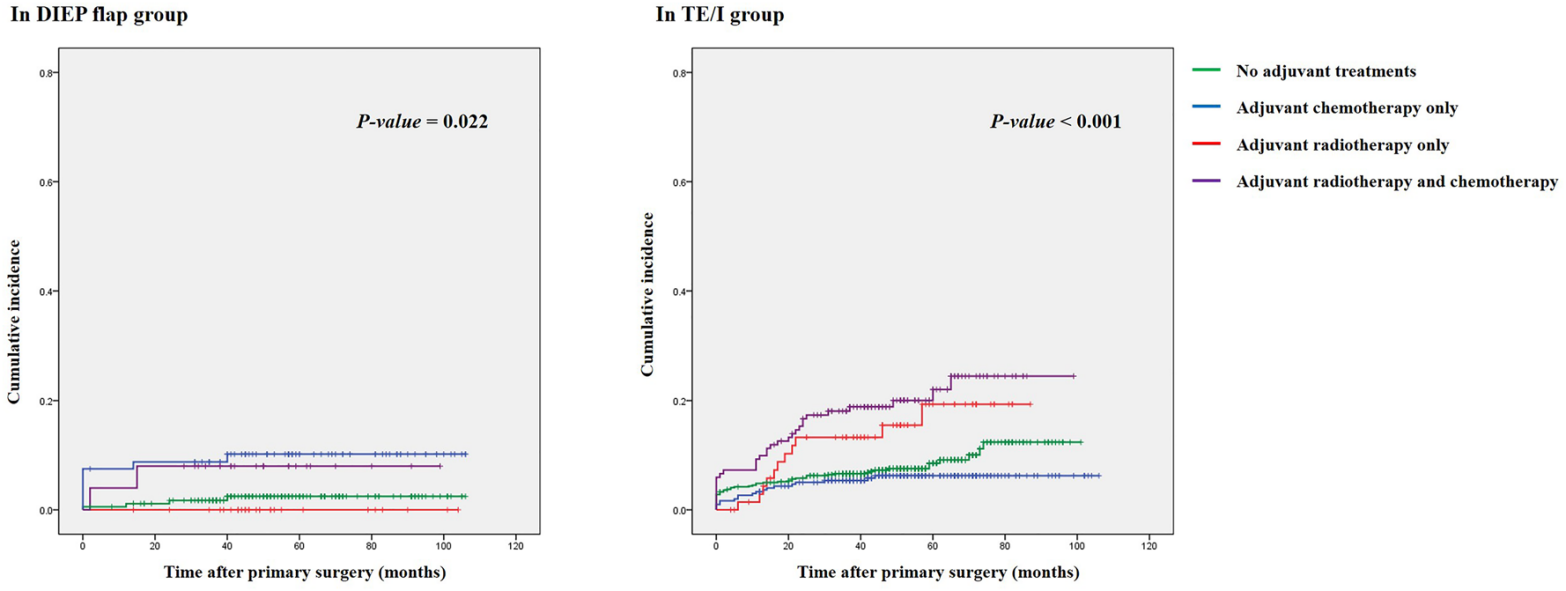
Cumulative incidence of major complications in the (left) deep inferior epigastric perforator (DIEP) and (right) tissue expander/implant (TE/I) groups.

The rate of major complications was evaluated in four subgroups categorized by adjuvant oncologic treatments: no adjuvant treatment, adjuvant chemotherapy only, adjuvant radiotherapy only, and adjuvant radiotherapy and chemotherapy. In the DIEP flap group, the two groups receiving adjuvant chemotherapy (adjuvant chemotherapy only and adjuvant radiotherapy and chemotherapy groups) showed significantly higher rates of the major complications than the other two groups (*p *= 0.020; see Supplementary Fig. S1). Similar results were observed in terms of the cumulative incidence of the major complications in the Kaplan–Meier analysis (see Fig. 3). Specifically, no differences were observed in the comparison of the cumulative incidences of the major complications between patients who had received adjuvant radiotherapy and those who had not (*p *= 0.753). However, significant differences were observed between those who had and had not received adjuvant chemotherapy (*p *= 0.002). In multivariate analyses, adjuvant chemotherapy, as well as hypertension and the weight of the mastectomy specimen showed a significant association with the development of the major complications in the DIEP flap group. Adjuvant radiotherapy was not associated with adverse outcomes.

#### In TE/I reconstruction

The rate of the major complications was significantly different according to adjuvant treatment, showing that the two groups receiving adjuvant radiotherapy (adjuvant radiotherapy only and adjuvant radiotherapy and chemotherapy groups) had a higher rate of adverse outcomes (see Supplementary Fig. S1). Consistent with the Kaplan–Meier analysis, patients receiving adjuvant chemotherapy and radiotherapy showed the highest cumulative incidence, followed by those receiving adjuvant radiotherapy only, while those receiving adjuvant chemotherapy showed the lowest cumulative incidence (*p *< 0.001; see Fig. 3). In contrast to the analyses for the DIEP flap group, comparison by adjuvant chemotherapy showed no difference (*p *= 0.288), while adjuvant radiotherapy showed a significant difference (*p *< 0.001). Multivariate Cox regression analyses demonstrated that adjuvant radiotherapy was significantly associated with the development of the major complications (HR, 2.612; 95% CI, 1.780–3.832; adjusted *p *< 0.001). A high BMI and the use of an acellular dermal matrix also showed significant associations. However, adjuvant chemotherapy did not show a significant association with adverse outcomes in this subgroup.

## Discussion

The present study evaluated the cumulative incidence of unexpected reoperation under general anesthesia and readmission following immediate breast reconstruction and compared it between cases using DIEP flaps and those using two-stage TE/I. The potential associations between reconstruction methods and the development of adverse outcomes were investigated in diverse clinical situations including conducting adjuvant treatments. Moreover, longitudinal analyses over a median follow-up period of approximately 5 years were performed in a considerable number of cases.

The main outcomes of interest in this study were unplanned reoperation under general anesthesia and/or unexpected readmission due to complications. These adverse events are the most serious complications that may considerably induce patient morbidity, tarnish the benefits of breast reconstruction, and affect oncologic outcomes, which should be considered primarily when selecting reconstruction methods for patients with breast cancer. Moreover, these events are never omitted from patient medical charts, which may minimize the potential risks of underestimation and overcome the inherent limitation of a retrospective study design to some degree.

In the current study, the two groups were heterogeneous in terms of several baseline characteristics including mastectomy type, weight of mastectomy specimen, BMI, and age. As mentioned above, we preferred to choose the option of DIEP flap for patients having higher BMI or large and ptotic breasts. This may contribute to generation of these heterogeneities. These variables have been reported to affect the outcomes of breast reconstruction. It is well known that patients undergoing nipple-sparing mastectomy may have a higher risk of adverse outcomes, including reconstruction failure, than those undergoing skin-sparing mastectomy, regardless of the reconstruction method^9^. In addition, several studies have reported that age may also influence the outcomes of breast reconstruction. Moreover, the impact of age on complications and patient satisfaction may differ according to the reconstruction method, showing that old age may have a negative influence on the outcomes of implant-based reconstruction but no influence on those of autologous reconstruction^10,11^. To minimize the confounding effects of heterogeneity between the groups, we conducted propensity score-matched analyses as well as multivariable logistic regression analyses.

In the long-term follow-up, we found that autologous tissue reconstruction using DIEP flaps was associated with a reduced risk of unexpected reoperation and/or readmission due to complications compared to two-stage TE/I-based reconstruction. These results were also supported by multivariate and propensity score matching analyses. Our findings are contradictory to those of previous studies with relatively short-term follow-ups. Jagsi et al. reported that overall wound complication rates were significantly higher in autologous reconstruction than in implant-based reconstruction within the first two postoperative years^12^. Bennett et al. reported similar outcomes, in that the use of DIEP flap for breast reconstruction was associated with approximately two-time higher odds of developing any complication than TE/I^8^. Autologous reconstruction may be more invasive with widely spanning operation fields and more complex in nature, and it can be rather apparent that it contains higher risks for complications during the early postoperative period. However, as time passes, the transferred autologous tissue can mature and gain longevity and stability with less vulnerability to external adverse impacts, such as radiation. This may lead to more favorable results for the DIEP flap over TE/I in the long run, as have previously reported. Fischer et al. reported that autologous tissue-based immediate reconstruction showed a significantly higher rate of 90-day complications and a lower rate of secondary breast procedures for three postoperative years than the TE/I method, which seems consistent with our findings^6^. It can be assumed that autologous tissue reconstruction may provide more reliable and safer outcomes than prosthetic reconstruction in the long run. However, in addition to postoperative complications, the potential risks of developing medical complications may need to be considered. In particular, deep vein thrombosis and pulmonary thromboembolism, although very rare, may be fatal for patients and may develop more frequently following DIEP flap-based reconstruction due to its long operation time and immobilization period. Also, we have found that the type of the major complications differed significantly between two groups, showing a higher rate of infection in the TE/I group and a higher rate of flap-related complications including total failure and fat necrosis in the DIEP flap group. This may contribute to generating significant differences in the timing of the adverse event development as well as in the rates of the major complications in the long-term follow-up.

We observed a more prominent association between TE/I and the development of major complications when restricting the analyses to individuals receiving adjuvant radiotherapy. This was further supported by additional subgroup analyses that showed that in cases using TE/I, receiving adjuvant radiotherapy was associated with an increased risk of developing major complications compared to the control, but was not associated with such a risk in those using DIEP flaps. Our findings are consistent with those of previous studies, which reported that implant-based reconstruction can be more vulnerable to the potentially adverse effects of radiation than autologous reconstruction^5,13,14^. In addition, postoperative radiotherapy was not a predictor of adverse outcomes in DIEP flap-based reconstruction. Based on these findings, it might be better for surgeons to inform patients who are expected to receive adjuvant radiotherapy of the potentially higher risks for unexpected reoperation/readmission that they are recommended to choose autologous tissue reconstruction if available.

In the present study, receiving adjuvant radiotherapy was found to be an independent risk factor for developing the major complications. Given that the rate of cases receiving it was slightly higher in the TE/I group than in the DIEP group, though not significantly different, a concern can be raised as to potential confounding effects of adjuvant radiotherapy on the outcomes. However, even in cases not receiving adjuvant treatments, the use of TE/I method was significantly associated with an increasing rate of the adverse outcome, compared to that of DIEP flap. This may suggest that a potential confounding effect related to the adjuvant radiotherapy may not influence the outcomes considerably.

Interestingly, adjuvant chemotherapy was associated with an increased risk of complications in DIEP flap-based reconstruction, but not in TE/I-based reconstruction. Previous studies investigating the potential influences of adjuvant chemotherapy on outcomes of DIEP flap-based breast reconstruction have been sparse^15^. Although adjuvant chemotherapy is usually delivered after wound healing has been completed, it could hinder the potential of wound healing and may lead to the development of delayed complications. Considering the wide-spanned operation fields and subsequently large-dimension wounds to be healed following DIEP flap-based breast reconstruction, higher complication rates in patients receiving adjuvant chemotherapy compared to the control may be plausible. In contrast, TE/I-based reconstruction has relatively narrow operation fields and less burden on wound healing; therefore, it may be less vulnerable to the potentially adverse effects of adjuvant chemotherapy. Based on these findings, patients who undergo DIEP flap-based breast reconstruction and are expected to receive adjuvant chemotherapy need to be informed of the potentially higher risks of unplanned reoperation/readmission after surgery.

The present study had several limitations. Its retrospective study design, with the subsequent risks of underestimation of the event, was an inherent limitation. All postoperative complications are significant issues for patients and may need to be evaluated to obtain more valid conclusions. However, This is challenging in retrospective studies; therefore, the current study evaluated only complications that resulted in unplanned reoperation under general anesthesia and/or unexpected readmission, which may limit the overall picture of the pros and cons of DIEP flap and TE/I reconstruction methods. In addition, although approximately 1,500 cases were included, the sample size based on a single institution may not be sufficient to draw concrete conclusions. An imbalanced sample size between the two groups (< 400 cases in the DIEP flap group) may have acted as a potential confounding factor in the statistical analysis. Lastly, this study population had relatively low BMIs and obesity rates compared to other populations, which may make it difficult to generalize our results. It has been established that obesity is an independent risk factor for postoperative complications, whose detrimental effects can be exaggerated in cases that have undergone operations with wide surgical fields, like DIEP flap-based breast reconstruction^16,17^. Therefore, conducting a similar analysis on obese patients with high BMIs may generate different results. Further large-scale multicenter studies are warranted to verify these results and generalize the conclusions.

## Conclusions

This 5-year longitudinal retrospective analysis revealed that autologous immediate breast reconstruction using a DIEP flap was associated with low rates of unexpected reoperation or readmission due to complications, with significantly lower odds of adverse events in the long run compared with two-stage TE/I-based reconstruction. This tendency toward favorable outcomes for DIEP flap-based reconstruction may differ according to adjuvant oncologic treatments and is more prominent in patients receiving adjuvant radiotherapy. Although further larger investigations with long-term follow-up are necessary, this information may be helpful in preoperative patient counseling to select a suitable breast reconstruction modality and postoperative care.

## Supplementary Information

Supplementary Legends.

Supplementary Figure S1.

Supplementary Tables.

## Data Availability

The datasets used and/or analyzed during the current study are available from the corresponding author upon reasonable request.
