# Supplementary material for: Longitudinal analysis of long-term outcomes of abdominal flap-based microsurgical reconstruction and two-stage prosthetic reconstruction
Source: Sci Rep. 2023 Mar 11;13:4062. doi: 10.1038/s41598-023-31218-2 (PMC10008543; doi:10.1038/s41598-023-31218-2)
Supplement: Supplementary file 1 — Supplementary Legends. [file 41598_2023_31218_MOESM1_ESM.pdf]

Supplementary Figure S1.
Incidence of major complications in each reconstruction group according to adjuvant treatment settings.
