# Supplementary figures and images for: Longitudinal analysis of long-term outcomes of abdominal flap-based microsurgical reconstruction and two-stage prosthetic reconstruction
Source: Sci Rep. 2023 Mar 11;13:4062. doi: 10.1038/s41598-023-31218-2 (PMC10008543; doi:10.1038/s41598-023-31218-2)

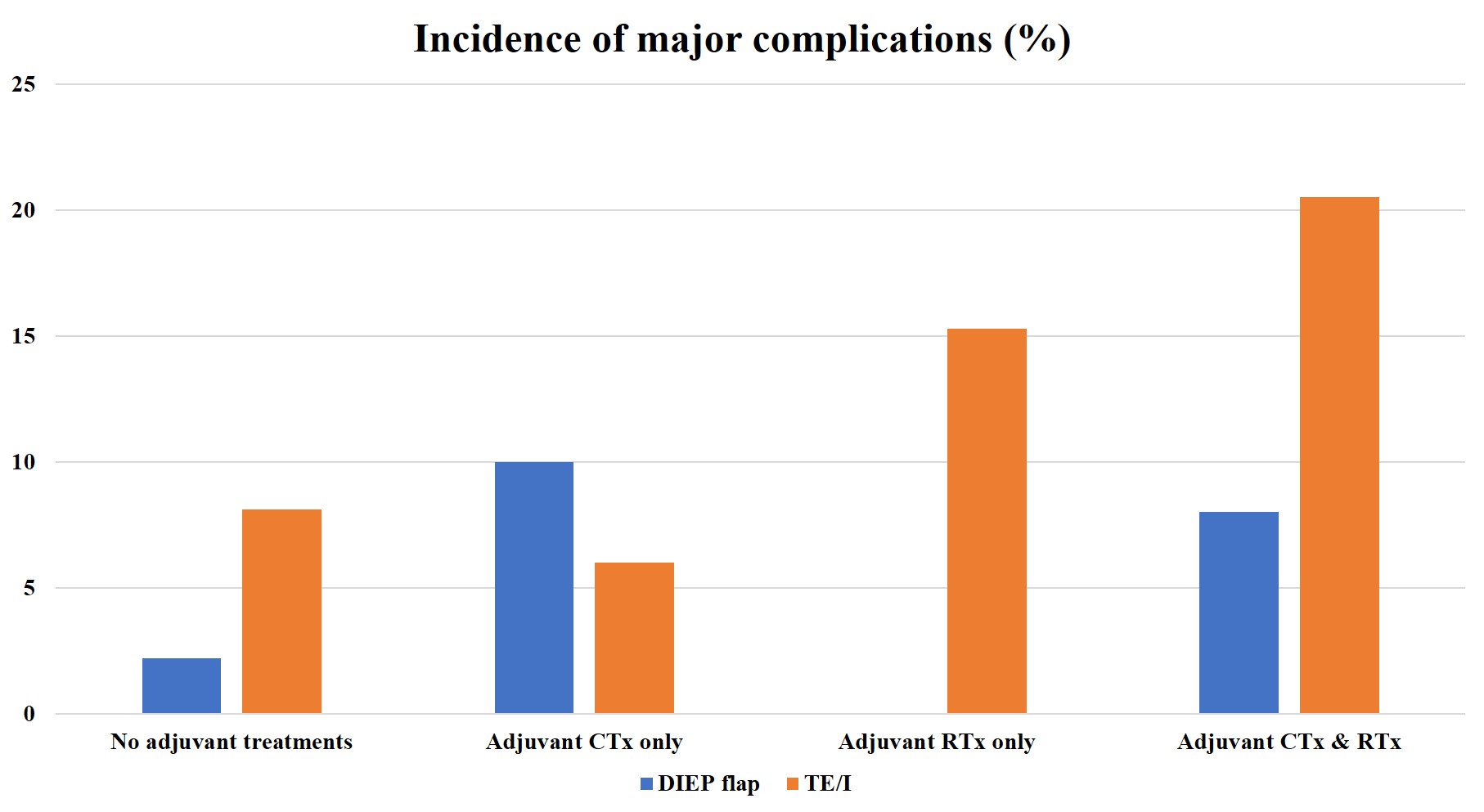

Supplement: Supplementary file 2 — Supplementary Figure S1. [file 41598_2023_31218_MOESM2_ESM.jpg]
