# Supplementary material for: Longitudinal analysis of long-term outcomes of abdominal flap-based microsurgical reconstruction and two-stage prosthetic reconstruction
Source: Sci Rep. 2023 Mar 11;13:4062. doi: 10.1038/s41598-023-31218-2 (PMC10008543; doi:10.1038/s41598-023-31218-2)
Supplement: Supplementary file 3 — Supplementary Tables. [file 41598_2023_31218_MOESM3_ESM.docx]

**Online Supplement Table 1.**

**Baseline characteristics of the two groups after propensity-score matching**

|  | TE/I group | DIEP flap group | p-value |
| --- | --- | --- | --- |
| Case No. | 312 | 312 |  |
| Age | 46.3 (± 7.2) | 46.3 (± 6.6) |  |
| BMI | 23.4 (± 3.0) | 23.6 (± 3.4) |  |
| Normal weight | 231 (74.0%) | 212 (67.9%) | 0.228 |
| Underweight | 6 (1.9%) | 9 (2.9%) |  |
| Overweight/Obesity | 75 (24.0%) | 91 (29.2%) |  |
| Diabetes | 3 (1.0%) | 5 (1.6%) | 0.477 |
| Hypertension | 27 (8.7%) | 25 (8.0%) | 0.772 |
| Smoking | 4 (1.3%) | 7 (2.2%) | 0.361 |
| Type of mastectomy |  |  | 0.088 |
| Nipple-sparing | 26 (8.3%) | 39 (12.5%) |  |
| Skin-sparing | 286 (91.7%) | 273 (87.5%) |  |
| Mastectomy weight | 468.7 (± 260.2) | 479.4 (± 205.9) |  |
| Neoadjuvant chemotherapy |  |  | 0.345 |
| Received | 27 (8.7%) | 34 (10.9%) |  |
| Not received | 285 (91.3%) | 278 (89.1%) |  |
| Adjuvant chemotherapy |  |  | 0.799 |
| Received | 102 (32.7%) | 105 (33.7%) |  |
| Not received | 210 (67.3%) | 207 (66.3%) |  |
| Adjuvant radiotherapy |  |  | 0.383 |
| Received | 46 (14.7%) | 54 (17.3%) |  |
| Not received | 266 (85.3%) | 258 (82.7%) |  |

**Online supplement Table 2.**

**Multivariable Cox regression analysis for the development of major complication in diverse clinical situations according to adjuvant oncologic treatments**

| Variables | HR (95% CI) | Adjusted p-value |
| --- | --- | --- |
| In cases with adjuvant radiotherapy |  |  |
| Diabetes | 4.659 (1.119 – 19.387) | 0.034 |
| Reconstruction methods |  | 0.016 |
| TE/I | Ref |  |
| DIEP flap | 0.175 (0.042 – 0.725) |  |
| In cases with adjuvant chemotherapy |  |  |
| Hypertension | 2.211 (0.991 – 4.936) | 0.053 |
| Mastectomy weight | 1.001 (1.000 – 1.002) | 0.040 |
| Adjuvant radiotherapy |  | < 0.001 |
| Not conducted | Ref |  |
| Conducted | 2.819 (1.668 – 4.763) |  |
| In cases with no adjuvant treatments |  |  |
| Age | 1.037 (1.001 – 1.075) | 0.044 |
| Type of mastectomy |  | 0.007 |
| Skin-sparing mastectomy | ref |  |
| Nipple-sparing mastectomy | 2.088 (1.220 – 3.571) |  |
| Mastectomy weight | 1.001 (1.000 – 1.002) | 0.012 |
| Reconstruction method |  | 0.009 |
| TE/I | Ref |  |
| DIEP flap | 0.255 (0.091 – 0.710) |  |
| Adjuvant radiotherapy |  | 0.006 |
| Not conducted | Ref |  |
| Conducted | 16.566 (2.216 – 123.852) |  |
